# Supplementary figures and images for: Upregulation of Metrnl improves diabetic kidney disease by inhibiting the TGF-β1/Smads signaling pathway: A potential therapeutic target
Source: PLoS One. 2024 Aug 27;19(8):e0309338. doi: 10.1371/journal.pone.0309338 (PMC11349091; doi:10.1371/journal.pone.0309338)

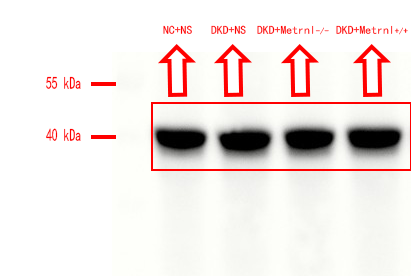

Supplement: S1 Raw images — (ZIP) [file pone.0309338.s001.zip › Original Images for BlotsGels/(a-sma)actin1 .tif]

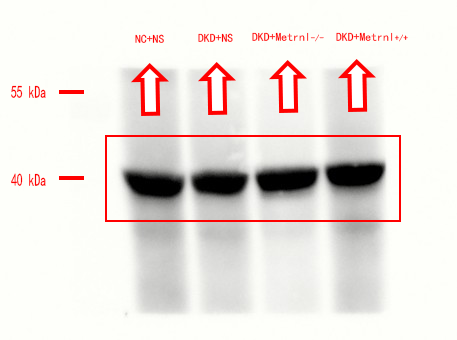

Supplement: S1 Raw images — (ZIP) [file pone.0309338.s001.zip › Original Images for BlotsGels/(┴φ╥╗╒┼═╝╡─)Actin.tif]

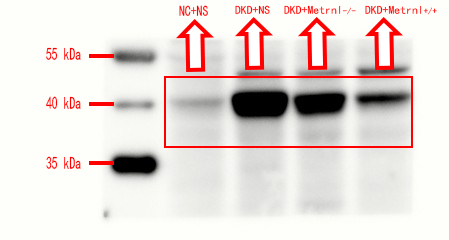

Supplement: S1 Raw images — (ZIP) [file pone.0309338.s001.zip › Original Images for BlotsGels/aSMA.tif]

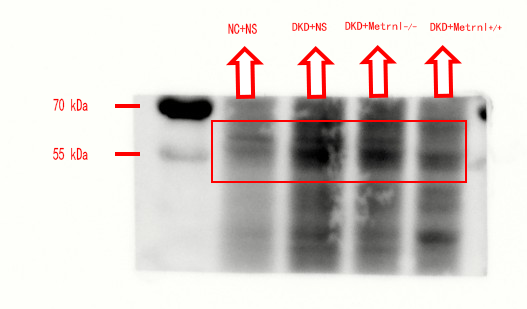

Supplement: S1 Raw images — (ZIP) [file pone.0309338.s001.zip › Original Images for BlotsGels/pSMA2.tif]

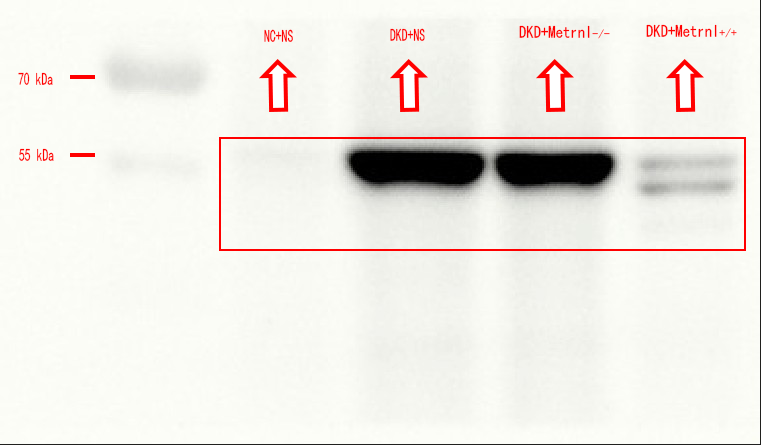

Supplement: S1 Raw images — (ZIP) [file pone.0309338.s001.zip › Original Images for BlotsGels/pSMA3.tif]

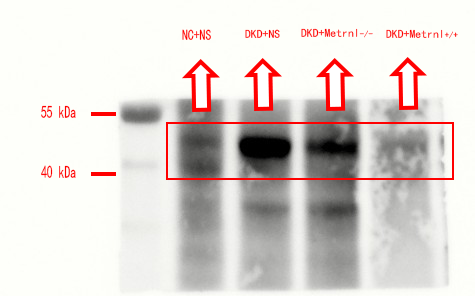

Supplement: S1 Raw images — (ZIP) [file pone.0309338.s001.zip › Original Images for BlotsGels/TGFR1.tif]

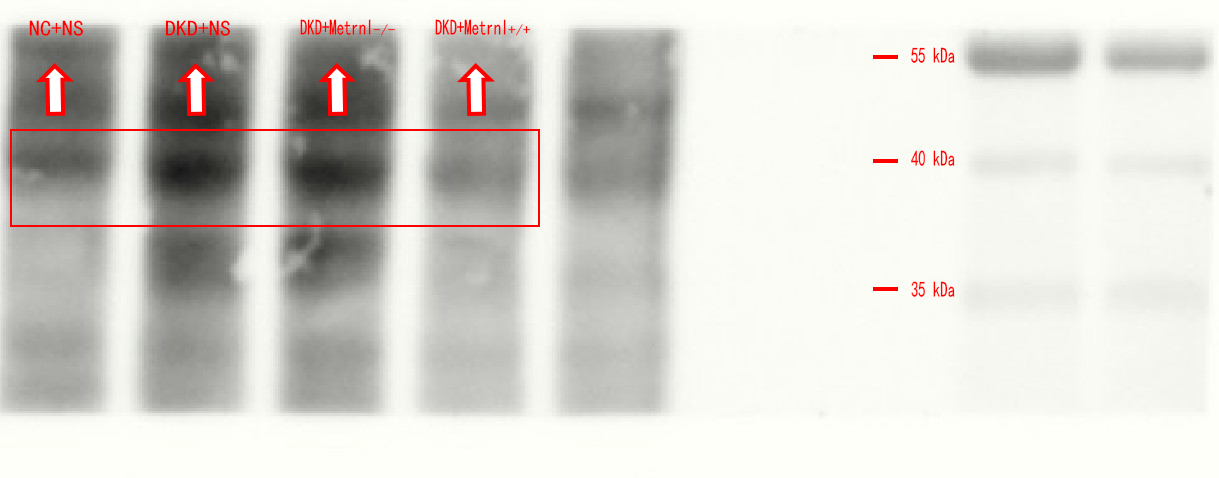

Supplement: S1 Raw images — (ZIP) [file pone.0309338.s001.zip › Original Images for BlotsGels/TGF-a┬1.tif]

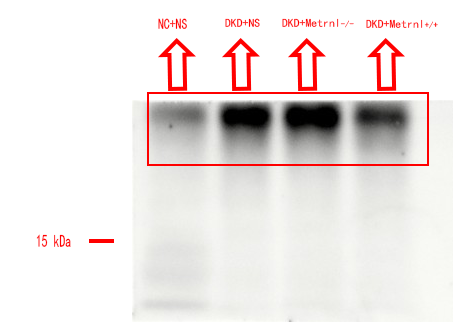

Supplement: S1 Raw images — (ZIP) [file pone.0309338.s001.zip › Original Images for BlotsGels/TNF-a┴.tif]

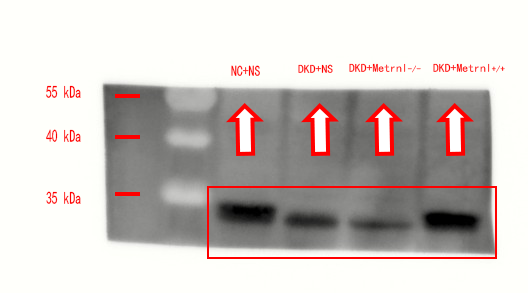

Supplement: S1 Raw images — (ZIP) [file pone.0309338.s001.zip › Original Images for BlotsGels/╖┼┤≤Metrnl.tif]

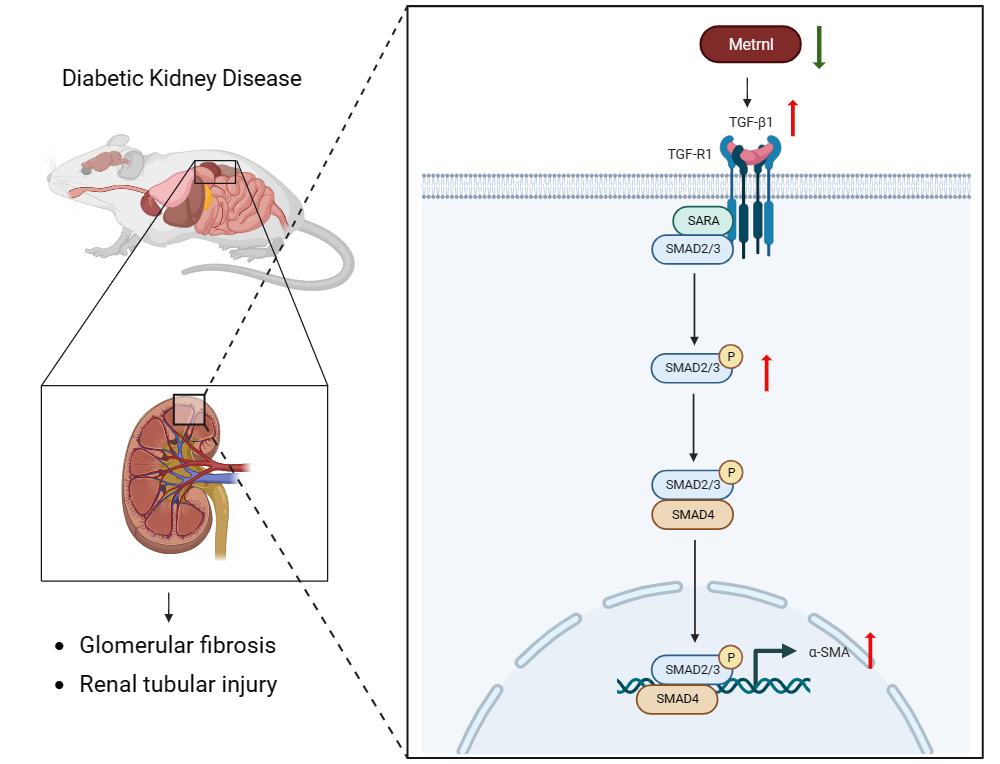

Supplement: S1 Graphical abstract — (PNG) [file pone.0309338.s003.png]
