# Supplementary material for: Upregulation of Metrnl improves diabetic kidney disease by inhibiting the TGF-β1/Smads signaling pathway: A potential therapeutic target
Source: PLoS One. 2024 Aug 27;19(8):e0309338. doi: 10.1371/journal.pone.0309338 (PMC11349091; doi:10.1371/journal.pone.0309338)
Supplement: S1 Checklist — (DOCX) [file pone.0309338.s004.docx]

*PLOS ONE* Humane Endpoints Checklist

*PLOS ONE* manuscript number: ___PONE-D-24-14449_______

**Complete the following if your study design includes death of a regulated animal as a likely outcome or planned experimental endpoint. Please also include all information in the Methods section of your manuscript.**

**ITEM 1.** **Describe whether humane endpoints* were used for all animals involved in the study.**

|  | **Recommendation** | **Section/Paragraph** |
| --- | --- | --- |
| **If humane endpoints* were used, report the following:** | | |
| **1** | **The specific criteria used to determine when animals should be euthanized** | **At the end of the animal experiment** |
| **2** | **Once animals reached endpoint criteria, the amount of time elapsed before euthanasia** | **Loss of consciousness, pain loss and relaxation, about 10 minutes** |
| **3** | **Whether any animals died before meeting criteria for euthanasia** | **There were four mice with high blood sugar in the long period of blood sugar, and the treatment of antiinfection was not effective in the inguoli area** |
| **If humane endpoints* were not used, report the following:** | | |
| **1** | **A scientific and ethical justification for the study design, including the reasons why humane endpoints could not be used, and discussion of alternatives that were considered but could not be used** |  |
| **2** | **Whether the institutional animal ethics committee specifically reviewed and approved the anticipated mortality in the study design** |  |

**ITEM 2.** **Include the following details of the study design and outcomes.**

|  | **Recommendation** | **Section/Paragraph** |
| --- | --- | --- |
| **1** | **The duration of the experiment** | **Twenty-one weeks** |
| **2** | **The numbers of animals used, euthanized, and found dead (if any); the cause of death for all animals** | **Twenty mice were euthanized at the end of the experiment, and four mice died after infection in the groin area, and were euthanized in advance** |
| **3** | **How frequently animal health and behavior were monitored** | **Weight, fasting blood glucose and morning urine microalbumin/creatinine ratio of mice in each group were measured every 4 weeks before the success of diabetic nephropathy model, and weight, fasting blood glucose and morning urine microalbumin/creatinine ratio of mice in each group were measured every day after the model was established** |
| **4** | **All animal welfare considerations taken, including efforts to minimize suffering and distress, use of analgesics or anaesthetics, or special housing conditions** | **Anaesthesia was induced by intraperitoneal injection of 1% sodium pentobarbital at a dose of 100 mg/kg to ensure humane euthanasia of the mice.** |
| **5** | **Any special training in animal care or handling provided for research staff** | **All researchers involved in animal testing have been professionally trained in animal feeding and euthanasia and have obtained animal testing certificates** |

***Definition of a humane endpoint**

A humane endpoint is an experimental endpoint at which animals are euthanized when they display early markers associated with death or poor prognosis of quality of life, or specific signs of severe suffering or distress. Humane endpoints are used as an alternative to allowing such conditions to continue or progress to death following the experimental intervention (“death as an endpoint”), or only euthanizing animals at the end of an experiment. Before a study begins, researchers define the practical observations or measurements that will be used during the study to recognize a humane endpoint, based on anticipated clinical, physiological, and behavioral signs. These may include, for instance, body temperature or weight changes, tumor size or appearance, abnormal behaviors, pathological changes, ruffled fur, reduced mobility, body posture, or expression of specific body fluid markers. Please see the NC3Rs guidelines for more information.

**ARRIVE Guidelines**

*PLOS ONE* encourages authors to follow the [Animal Research: Reporting of In Vivo Experiments (ARRIVE) guidelines](http://www.nc3rs.org.uk/arrive-guidelines) for all submissions describing laboratory-based animal research and to upload a completed [ARRIVE Guidelines Checklist](http://www.nc3rs.org.uk/sites/default/files/documents/Guidelines/NC3Rs%20ARRIVE%20Guidelines%20Checklist%20%28fillable%29.pdf) to be published as supporting information.
