# Supplementary material for: Upregulation of Metrnl improves diabetic kidney disease by inhibiting the TGF-β1/Smads signaling pathway: A potential therapeutic target
Source: PLoS One. 2024 Aug 27;19(8):e0309338. doi: 10.1371/journal.pone.0309338 (PMC11349091; doi:10.1371/journal.pone.0309338)
Supplement: Attachment — Submitted filename: Dear respected editor.docx [file pone.0309338.s006.docx]

 Dear respected editor,

I hope you are fine.

Thank you for giving me this opportunity to review the paper entitled: **"Up-regulation of Metrnl improves diabetic kidney disease by inhibiting the TGF-**1 **β1/Smads signaling pathway: A potential therapeutic target”.** This paper aimed to explore the relationship between Metrnl and DKD. The topic is interesting. However, the integrity and quality of the manuscript is poor and can improve by considering the comments pointed out below.

 Comments:

1. The manuscript would need a partial review of the English language. The presenting content has minor spelling and grammatical errors, especially in the abstract.
2. Some of the sentences have no references, please revise and add their references. For example: "Several studies have examined the association between Metrnl level and adverse diabetic renal events in patients with DKD. These studies found a negative correlation between serum Metrnl concentration and the risk of DKD", Ref?
3. In the purpose section of the abstract, "This study aimed to investigate the role of Metrnl in this model" What do you mean by "this"?
4. It is suggested to add a box of abbreviations before the abstract, many of the abbreviations in the abstract are incomprehensible.
5. The mechanism of action of Metrnl in the control of diabetic kidneys is better depicted in a figure or graphical abstract.
6. Some explanations in the discussion are not needed. Please add the results of previously assessed studies and justify the obtained results.
