# Supplementary material for: Upregulation of Metrnl improves diabetic kidney disease by inhibiting the TGF-β1/Smads signaling pathway: A potential therapeutic target
Source: PLoS One. 2024 Aug 27;19(8):e0309338. doi: 10.1371/journal.pone.0309338 (PMC11349091; doi:10.1371/journal.pone.0309338)
Supplement: Attachment — Submitted filename: Response to Reviewers.doc [file pone.0309338.s007.doc]

Editor-in-Chief

PLOS ONE

Dear Editor:

We wish to re-submit our manuscript, titled “Upregulation of Metrnl improves diabetic kidney disease by inhibiting the TGF-β1/Smads signaling pathway: A potential therapeutic target” The manuscript ID is PONE-D-24-14449R1.

We thank you and the reviewers for your thoughtful suggestions and insights. The manuscript has benefited from these insightful suggestions. I look forward to working with you and the reviewers to move this manuscript closer to publication in PLOS ONE.

The manuscript has been rechecked, and the necessary changes have been made in accordance with the reviewers’ suggestions. The responses to all comments have been prepared and attached herewith. We have also ensured that the references are closely related to the content of this study.

Thank you for your consideration. I look forward to hearing from you.

Sincerely,

Pin Chen,

Email address: chenpin@21cn.com

**COMMENTS TO THE AUTHOR:**

**Reviewer :**

1. **The manuscript would need a partial review of the English language. The presenting content has minor spelling and grammatical errors, especially in the abstract.**

**Response:** Thank you for your valuable feedback. I have thoroughly reviewed and corrected the manuscript, particularly focusing on the spelling and grammatical errors you pointed out, especially in the abstract. The language has been refined to ensure clarity and accuracy throughout the manuscript.

2. **Some of the sentences have no references, please revise and add their references. For example: "Several studies have examined the association between Metrnl level and adverse diabetic renal events in patients with DKD. These studies found a negative correlation between serum Metrnl concentration and the risk of DKD", Ref?**

**Response:** Thank you for your insightful comments. I have revised the manuscript to include the necessary references for the sentences that previously lacked citations (marked in red, lines 41-42, 50-53, 63-65, 392-394, 406-407, 425-426. reference1, 8, 14, 22, 23, 26). The references have been carefully selected to support the claims made and ensure the accuracy and credibility of the manuscript.

3. **In the purpose section of the abstract, "This study aimed to investigate the role of Metrnl in this model" What do you mean by "this"?**

**Response:** Thank you for pointing out the need for clarification. In the purpose section of the abstract, "this model" refers to the specific experimental model used in the study to investigate the role of Metrnl. I have revised the sentence for better clarity: "This study aimed to investigate the role of Metrnl in an experimental model of diabetic kidney disease." (marked in red, lines 19-20)

4. **It is suggested to add a box of abbreviations before the abstract, many of the abbreviations in the abstract are incomprehensible.**

**Response:** Thank you for your helpful suggestion. I have added a box of abbreviations before the abstract to ensure that all abbreviations used are clearly explained and comprehensible to the readers (marked in red, lines 12-18).

5. **The mechanism of action of Metrnl in the control of diabetic kidneys is better depicted in a figure or graphical abstract.**

**Response:** Thank you for your suggestion. I agree that a figure or graphical abstract would better depict the mechanism of action of Metrnl in the control of diabetic kidney disease. I have included a graphical abstract that visually represents the key mechanisms and findings of the study (Graphical abstract).

6. **Some explanations in the discussion are not needed. Please add the results of previously assessed studies and justify the obtained results.**

**Response:** Thank you for your valuable feedback. I have revised the discussion section to include the results of previously assessed studies. Additionally, I have provided a justification for the obtained results in the context of existing literature. This revision aims to enhance the discussion by linking our findings to the broader body of research and providing a comprehensive understanding of the study's implications.

7. **In addition to above, could you please clarify how you generated the Supp. fig. 2 (mouse with organ)**

**Response:** Thank you for your question regarding the generation of Supplementary Figure 2. The figure of the mouse with organs was created using the BioRender website. The entire image is original and designed specifically for this study.

**END**
